# Predictors of outcome in large vessel occlusion stroke patients with intravenous tirofiban treatment: a post hoc analysis of the RESCUE BT clinical trial

**DOI:** 10.1186/s12883-024-03733-w

**Published:** 2024-07-01

**Authors:** Xiang Liu, Wencheng He, Meiqiong Li, Jie Yang, Jiacheng Huang, Weilin Kong, Changwei Guo, Jinrong Hu, Shuai Liu, Dahong Yang, Jiaxing Song, Zhouzhou Peng, Linyu Li, Yan Tian, Wenjie Zi, Chengsong Yue, Fengli Li

**Affiliations:** 1 Department of Neurology, The Second Affiliated Hospital, Xinqiao Hospital, Army Medical University, Third Military Medical University, Chongqing, 400037 China; 2 Department of Neurology, Guangxi Guiping People’s Hospital, Guiping, Guangxi China

**Keywords:** Tirofiban, Acute large vessel occlusion stroke, Endovascular thrombectomy, Forecasting

## Abstract

**Objective:**

The aim of this study was to investigate the factors influencing good outcomes in patients receiving only intravenous tirofiban with endovascular thrombectomy for large vessel occlusion stroke.

**Methods:**

Post hoc exploratory analysis using the RESCUE BT trial identified consecutive patients who received intravenous tirofiban with endovascular thrombectomy for large vessel occlusion stroke in 55 comprehensive stroke centers from October 2018 to January 2022 in China.

**Results:**

A total of 521 patients received intravenous tirofiban, 253 of whom achieved a good 90-day outcome (modified Rankin Scale [mRS] 0–2). Younger age (adjusted odds ratio [aOR]: 0.965, 95% confidence interval [CI]: 0.947–0.982; *p* < 0.001), lower serum glucose (aOR: 0.865, 95%CI: 0.807–0.928; *p* < 0.001), lower baseline National Institutes of Health Stroke Scale (NIHSS) score (aOR: 0.907, 95%CI: 0.869–0.947; *p* < 0.001), fewer total passes (aOR: 0.791, 95%CI: 0.665–0.939; *p* = 0.008), shorter punctures to recanalization time (aOR: 0.995, 95%CI:0.991–0.999; *p* = 0.017), and modified Thrombolysis in Cerebral Infarction (mTICI) score 2b to 3 (aOR: 8.330, 95%CI: 2.705–25.653; *p* < 0.001) were independent predictors of good outcomes after intravenous tirofiban with endovascular thrombectomy for large vessel occlusion stroke.

**Conclusion:**

Younger age, lower serum glucose level, lower baseline NIHSS score, fewer total passes, shorter punctures to recanalization time, and mTICI scores of 2b to 3 were independent predictors of good outcomes after intravenous tirofiban with endovascular thrombectomy for large vessel occlusion stroke.

**Chinese clinical trial registry identifier:**

ChiCTR-IOR-17014167.

**Supplementary Information:**

The online version contains supplementary material available at 10.1186/s12883-024-03733-w.

## Introduction

Endovascular treatment has become a prerequisite for successful reperfusion of acute intracranial large vessel occlusive stroke, including mechanical thrombectomy of retrievable stents, contact suction thrombectomy, balloon and/or stent implantation, and intra-arterial thrombolysis. However, studies have shown that 10–15% of patients cannot achieve successful recanalization after endovascular thrombectomy [1]. Injury to blood vessels and exposure to the subendothelial matrix after thrombectomy, which result in platelet activation, adhesion, and aggregation, are the main causes of acute and delayed reocclusion of blood vessels [2, 3].

Platelet glycoprotein IIb/IIIa receptor antagonists inhibit platelet aggregation by blocking the binding of fibrinogen or von Willebrand factor to platelet glycoprotein IIb/IIIa receptors [4]. Given the advantages of tirofiban, many studies have investigated its combination in the intravascular treatment of acute ischemic stroke; however, most of them were single-center and small-sample observational studies [5–7]. Although the RESCUE BT study showed that tirofiban did not significantly alter the outcome at 90 days after endovascular thrombectomy for large vessel occlusive stroke [8], tirofiban is still commonly used for rescue therapy and thrombosis during the perioperative period. Therefore, determining which patients are likely to achieve the greatest benefit from tirofiban is of great value and necessitates further research.

Currently, few studies have explored the characteristics of patients who benefit from endovascular thrombectomy combined with intravenous tirofiban for large vessel occlusive stroke. Therefore, we aimed to explore the clinical characteristics of patients who benefit from endovascular thrombectomy for large vessel occlusive stroke combined with intravenous tirofiban through a subgroup analysis of the RESCUE BT trial.

## Method

### Study design

This was a post hoc analysis of the RESCUE BT trial which was registered on the Chinese Clinical Trial Registry (ChiCTR-IOR-17,014,167). The study was approved by the ethics committee of the Xinqiao Hospital, Army Medical University, and all participating centers, participation in the study and publication was informed consent for all patients.

We used data from the RESCUE BT trial, an investigator-initiated, multicenter, randomized, double-blind, placebo-controlled trial [8]. The complete protocol of the RESCUE BT study has been described elsewhere [9]. For the present study, we selected patients with the following criteria: (1) treated with tirofiban; (2) aged ≥ 18 years; (3) occlusion of the intracranial internal carotid artery, or the first or second segment of the middle cerebral artery confirmed by CT, MR angiography, or digital subtraction angiography. We excluded patients who used a placebo but did not include the rescue use of tirofiban. Patients with an mRS score of 0–2 at 90 days were defined as the good outcome group, and those with an mRS score of 3–6 at 90 days were defined as the poor outcome group.

### Measures and outcomes

Demographic data, medical history, clinical data, and procedural characteristics were collected for analysis. The baseline NIHSS score was used to assess stroke severity at baseline, and the Baseline Alberta Stroke Program Early CT Score (ASPECTS) was used to quantify the infarct core before treatment [10]. Successful recanalization was defined as an mTICI score of 2b (50–99% reperfusion) to 3 (complete reperfusion) [11]. Subsequently, we constructed a predictive model to evaluate the probability of a good outcome by screening the influencing factors through multivariable logistic regression analysis. The safety outcome was symptomatic intracranial hemorrhage (sICH), which was determined according to the Heidelberg Bleeding Classification [12].

### Statistical methods

SPSS (version 26, IBM Corp.), MedCalc (version 20.0.22), and R (version 4.2.1, R Foundation for Statistical Computing) software were used. Data are expressed as numbers (percentages), means (SD), or medians (interquartile ranges). Differences between patients with good and poor outcomes were assessed using the Mann–Whitney U test or Student’s t-test for continuous variables and the Pearson chi-square test or Fisher’s exact test for categorical variables. The final influencing factors of good outcomes were determined using multivariable regression analysis of single-factor analysis *p* < 0.1. The aOR with 95%CI was reported in the model. The variance inflation factor (VIF) was used to evaluate the collinearity between various factors [13]. A predictive model was developed based on these factors. The Hosmer–Lemeshow goodness-of-fit test, C-index, and calibration curve were used to evaluate the predictive model. Decision curve analysis (DCA) was performed to assess the clinical usefulness of the predictive model for good outcomes. The mediating effects of sICH were analyzed. Furthermore, the receiver operating characteristic (ROC) curve and Youden index were used to determine cutoff values for continuous variables. All statistical tests of the hypotheses were two-sided, and P-values < 0.05 were considered significant.

## Results

In the RESCUE BT trial, 521 patients received intravenous tirofiban and 58 patients received tirofiban as a rescue drug. A total of 253 (48.6%) patients achieved a good outcome and 268 (51.4%) patients had a poor outcome. Compared to the poor outcome, younger age (median, 64.0 versus 70.0 years; *p* < 0.001), male (64.0% versus 53.0%, *p* = 0.014), lower baseline NIHSS score (median, 13.0 versus 17.0, *p* < 0.001), baseline ASPECTS (median, 8.0 versus 8.0, *p* = 0.042), lower systolic blood pressure (SBP; mean, 143.3 versus 149.2 mmHg; *p* = 0.006), lower serum glucose (median, 6.4 versus 7.4 mmol/L; *p* < 0.001), absence of hypertension (48.2% versus 61.6%; *p* = 0.003), absence of diabetes mellitus (14.6% versus 28.7%; *p* < 0.001), shorter puncture to recanalization time (median, 61.0 versus 73.0 min; *p* = 0.010), reduced number retriever of total passes (median, 1.0 versus 2.0; *p* = 0.001), and mTICI score 2b to 3 (98.0% versus 87.3%; *p* < 0.001) were shown to be predictive of a better outcome. The other characteristics were not statistically significant and the baseline characteristics are shown in Table 1. The VIF for all factors was < 2 (Supplementary Table S1). The characteristics of patients with sICH are shown in Supplementary Table S2.

**Table 1:** Baseline characteristics of patients of Intravenous Tirofiban Before Endovascular Thrombectomy

	Good outcome	Poor outcome	*P* value
Characteristic	mRS 0–2	mRS 3–6	
No. of patients	253	268	
Age, median (IQR), y	64.0 (54.0, 72.0)	70.0 (61.0, 76.0)	< 0.001
Sex			0.014
Female, no. (%)	91 (36.0)	126 (47.0)	
Male, no. (%)	162 (64.0)	142 (53.0)	
Baseline NIHSS, median (IQR)	13.0 (10.0, 18.0)	17.0 (13.0, 20.0)	< 0.001
Baseline ASPECTS, median (IQR)	8.0 (7.0, 9.0)	8.0 (6.0, 9.0)	0.042
SBP, mean (SD), mm Hg	143.3 (22.9)	149.2 (25.5)	0.006
DBP, median (IQR), mm Hg	82.0 (75.0, 91.0)	85.0 (75.0, 95.0)	0.066
Serum glucose, median (IQR), mmol/L	6.4 (5.4, 7.7)	7.4 (6.2, 9.1)	< 0.001
Vascular risk factor			
Coronary heart disease, no. (%)	32 (12.6)	43 (16.0)	0.328
Atrial fibrillation, no. (%)	77 (30.4)	93 (34.7)	0.345
Hypertension, no. (%)	122 (48.2)	165 (61.6)	0.003
Hyperlipidemia, no. (%)	37 (14.6)	46 (17.2)	0.502
Diabetes mellitus, no. (%)	37 (14.6)	77 (28.7)	< 0.001
Ischemic stroke, no. (%)	33 (13.0)	44 (16.4)	0.336
Smoking, no. (%)	61 (24.1)	55 (20.5)	0.38
Prestroke mRS score, no. (%)			0.307
0	240 (94.9)	245 (91.4)	
1	7 (2.8)	15 (5.6)	
2	6 (2.4)	7 (2.6)	
4	0 (0.0)	1 (0.4)	
Stroke etiology, no. (%)			0.73
LAA	122 (48.2)	129 (48.1)	
CE	106 (41.9)	107 (39.9)	
Other causes	25 (9.9)	32 (11.9)	
Occlusion sites, no. (%)			0.11
Intracranial ICA	41 (16.2)	63 (23.5)	
M1 middle cerebral artery segment	178 (70.4)	174 (64.9)	
M2 middle cerebral artery segment	34 (13.4)	31 (11.6)	
Onset to puncture time, min, median (IQR)	409.0 (250.0, 639.0)	427.0 (291.0, 651.2)	0.229
Puncture to recanalization time, min, median (IQR)	61.0 (40.0, 97.0)	73.0 (45.0, 122.2)	0.01
Total passes^a^, median (IQR)	1.0 (1.0, 2.0)	2.0 (1.0, 3.0)	0.001
mTICI score 2b to 3, no. (%)	248 (98.0)	234 (87.3)	< 0.001

Abbreviations: NIHSS, National Institutes of Health Stroke Scale; ASPECTS, Acute Stroke Prognosis Early Computed Tomography Score; SBP, systolic blood pressure; DBP, diastolic blood pressure; mRS, modified Rankin Scale; LAA, large artery atherosclerosis; CE, cardioembolism; mTICI, modified Thrombolysis in Cerebral Infarction score 2b (50–99% reperfusion) to 3 (complete reperfusion)

^a^ The number of retriever total passes

**Fig. 1:**
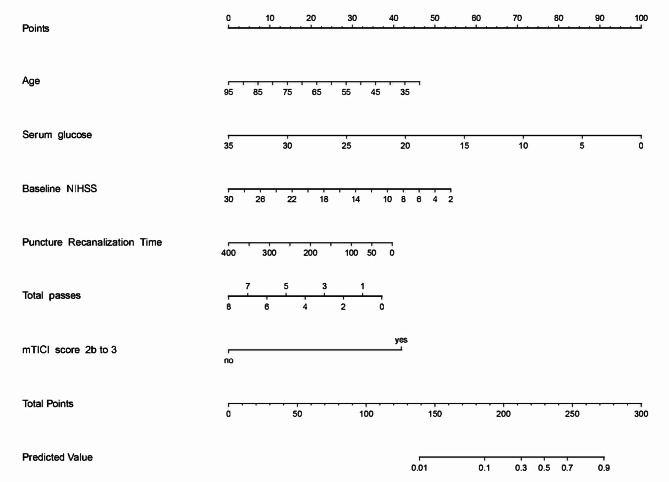
The nomogram for predicting a good outcome in patients with only intravenous tirofiban with endovascular thrombectomy for large vessel occlusion stroke. For each patient, we added the scores of the six influencing factors corresponding to the “Points”, and then the prediction results were obtained based on the predicted values corresponding to the “Total Points”. NIHSS, National Institutes of Health Stroke Scale; Total passes, the total number of passes of retriever; mTICI, modified Thrombolysis in Cerebral Infarction score 2b (50–99% reperfusion) to 3 (complete reperfusion)

Multivariate regression analysis revealed independent influencing factors for good outcomes (Table 2). A predictive model was constructed using these influencing factors and is presented as a nomogram (Fig. 1). In the multivariate regression analysis of sICH, serum glucose (aOR: 1.107, 95%CI: 1.029–1.191; *p* = 0.007), platelet count (aOR: 0.989, 95%CI: 0.983–0.996; *p* = 0.001), and baseline ASPECTS (aOR: 0.817, 95%CI: 0.681–0.981; *p* = 0.031) were independent risk factors for sICH (Table 2). Simultaneously, the baseline ASPECTS affected the outcome through sICH (Figure S1 in the Data Supplement). At the same time, we performed outcome-influencing factors analysis on 427 patients in the non-Tirofiban group excluding 58 patients who received rescue tirofiban therapy (Table S3 and Table S4). And constructed an outcome-prediction model and nomogram based on these findings (Figure S3 in the Data Supplement).

**Table 2:** Multivariable analysis: predictors of a good outcome and sICH

Variable	aOR (95% CI)	*p* Value
Age	0.965(0.947–0.982)	< 0.001
Serum glucose	0.865(0.807–0.928)	< 0.001
Baseline NIHSS	0.907(0.869–0.947)	< 0.001
Total passes	0.791(0.665–0.939)	0.008
Puncture to Recanalization Time	0.995(0.991–0.999)	0.017
mTICI score 2b to 3	8.330(2.705–25.653)	< 0.001
sICH		
Serum glucose	1.107(1.029–1.191)	0.007
Pretreatment platelet count	0.989(0.983–0.996)	0.001
Baseline ASPECTS	0.817(0.681–0.981)	0.031

**Fig. 2:**
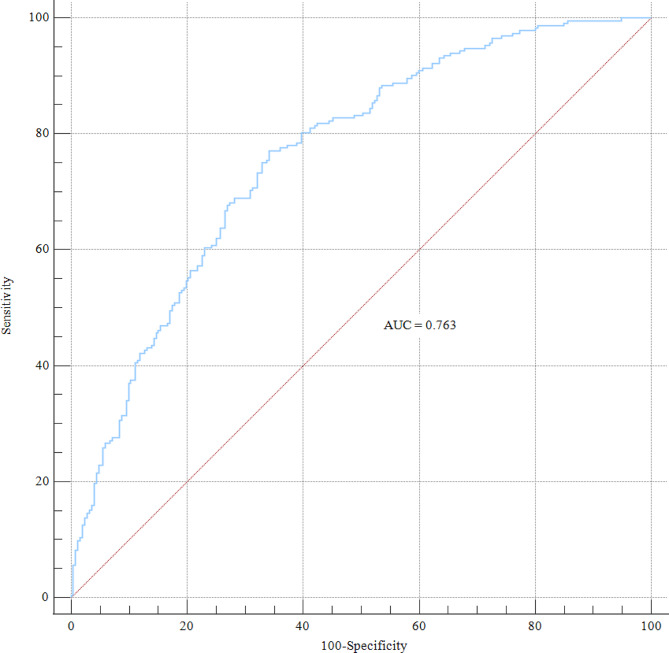
The ROC curve of the present prediction model for a good outcome with intravenous tirofiban with endovascular thrombectomy for large vessel occlusion stroke. AUC, the area under the curve; ROC, receiver operating characteristic

**Fig. 3:**
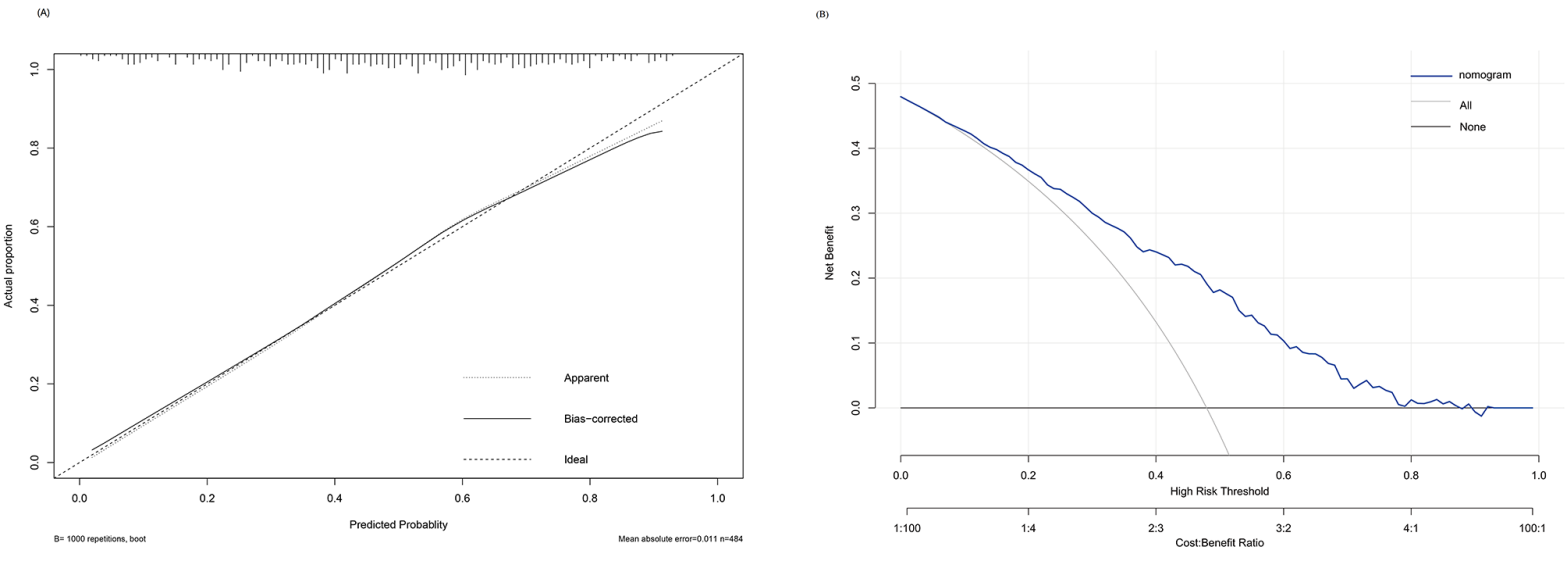
**A**, the calibration curves of the good outcome nomogram. The x-axis represents the predicted value with a good outcome in intravenous tirofiban with endovascular thrombectomy for large vessel occlusion stroke. The y-axis represents the actual proportion. The diagonal dashed line was a perfect prediction by an ideal model. The dotted line is the performance of the nomogram of a good outcome, while the solid line corrects for any bias in the nomogram. **B**, Decision curve analysis for the nomogram in the intravenous tirofiban with endovascular thrombectomy for large vessel occlusion stroke. If the threshold probability of a patient was > 1 and < 69%, application of the nomogram of good outcome would add more net benefit to patients

We calculated the C-index to investigate the nomogram, which was 0.763 (95%CI: 0.722–0.800), and the AUROC was 0.763 (Fig. 2). The calibration curve also showed that the nomogram had good prediction accuracy (Fig. 3A). The DCA of a good outcome nomogram indicates that it can provide higher net benefits (Fig. 3B) and has good clinical application value if the threshold probability range is approximately 1–69%.

We further determined the cut-off for continuous variables using the ROC curve and the Youden index. Age ≤ 68 years (AUC = 0.622, Youden index 0.197, 95%CI: 0.579–0.664, *p* < 0.001), baseline NIHSS score ≤ 13 (AUC = 0.648, Youden index 0.238, 95%CI: 0.605–0.689, *p* < 0.001), serum glucose ≤ 6.89 mmol/L (AUC = 0.639, Youden index 0.227, 95%CI: 0.594–0.682, *p* < 0.001), total passes ≤ 2 (AUC = 0.583, Youden index 0.134, 95%CI: 0.539–0.626, *p* < 0.001), and puncture to recanalization time ≤ 89 min (AUC = 0.565, Youden index 0.115, 95%CI: 0.521–0.608, *p* = 0.010) were shown to be associated with achieve a good outcome (Figure S2 in the Data Supplement).

## Discussion

In this study, we explored the factors affecting the good outcomes at 90 days and the safety outcomes of tirofiban with endovascular thrombectomy for large vessel occlusive stroke using multivariable regression analysis. We constructed a predictive model to predict the probability of a good outcome based on age, serum glucose, baseline NIHSS score, total passes, puncture to recanalization time, and an mTICI score of 2b to 3. The possibility of a good outcome increases steeply with an increase in the total points. The calibration curve and C-index indicated that the predictive model had good discrimination and calibration abilities.

We also determined the clinical implications of our nomogram. First, as there is a lack of clinical predictive models for good outcomes with tirofiban in the endovascular treatment of large-vessel occlusive stroke, the model provides a reference for analyzing the outcomes of patients using tirofiban. Second, these results further emphasize the importance of serum glucose, total passes, puncture to recanalization time, and mTICI scores of 2b to 3 to improve functional recovery. Thus, the nomogram may represent the optimal model for predicting a good outcome for tirofiban combined with endovascular treatment in large vessel occlusive stroke; if so, this will serve to help neurologists make personalized clinical decisions (e.g., shortening the puncture-to-recanalization time) to further improve outcomes.

Comparing these factors for the group with good outcomes showed that serum glucose was a cofactor of sICH. Previous studies have shown that hyperglycemia is associated with poor prognosis after endovascular thrombectomy [14, 15]. Indeed, a multicenter study based on the Japanese population showed that admission hyperglycemia with or without endovascular thrombectomy was associated with sICH [16]. These previous results are consistent with our findings and may be explained by the fact that many studies have shown that hyperglycemia is associated with an increased cerebral infarction volume and causes a poor outcome [17–19]. The infarct volume has been shown to have a good correlation with ASPECTS [20], and in our study, multivariate analysis of sICH also showed that baseline ASPECTS was an independent factor and indirectly affected the good outcome. Passes of an intravascular thrombectomy device can easily damage the blood-brain barrier and microvessels, and hyperglycemia increases the risk of sICH and a poor outcome [21, 22]. It is worth noting that when the number of retrievers was ≤ 2 the benefit was better in our study. Indeed, previous studies have shown that a higher number of passes with a thrombectomy device increases the risk of death, even in patients with recanalization [23]. Besides, hyperglycemia may damage vascular integrity by inducing excitatory chemokines and oxidative stress, attacking neurovascular units, and promoting edema and other biological mechanisms [24]. In the good outcome group of this study, the serum glucose median was 6.4 (interquartile range: 5.4, 7.7) mmol/L, and a serum glucose level ≤ 6.89 mmol/L may achieve a better outcome for tirofiban with endovascular treatment in large vessel occlusive stroke. However, a subgroup analysis of MR CLEAN showed that serum glucose at admission did not affect the outcome of endovascular treatment of acute ischemic stroke [25]. Unfortunately, we were unable to confirm whether a better prognosis could be obtained after controlling serum glucose and improving safety outcomes. Therefore, it is necessary to design a multicenter randomized controlled trial of tirofiban to guide the control of serum glucose levels.

Additionally, our results showed that the incidence of sICH (9.4%) was similar to that reported in the North American Solitaire Stent Retriever Acute Stroke Registry (9.9%) [26]. In real-world practices, a higher risk of sICH may result from expanded indications, insufficient experiences, or unfavorable treatment conditions. Moreover, a previous observational study demonstrated that the use of tirofiban was associated with sICH [27]. Furthermore, in the safety outcome analysis, the platelet count was also associated with sICH. Tirofiban prevents platelet aggregation by inhibiting the platelet glycoprotein IIb/IIIa receptor [28]. Some studies have shown that the incidence of further platelet reduction caused by tirofiban is 0.5-5.6% [29]. Therefore, tirofiban should be used with caution in patients with thrombocytopenia who have undergone endovascular thrombectomy for large-vessel occlusive stroke.

In this study, the mTICI score 2b to 3 was identified as a factor affecting the good outcomes. Yang et al [30]. believed that the intravenous use of tirofiban in endovascular thrombectomy could improve the recanalization rate and prognosis. Some studies have suggested that the rich collateral circulation may explain the high recanalization rate of patients with intravenous tirofiban [31, 32]. Tirofiban dissolves distal thrombosis through good collateral circulation to reduce the probability of reocclusion [30]. In patients with acute large-vessel occlusion, patients with good collateral circulation may see a greater benefit of tirofiban.

We found that the time from stroke onset to groin puncture was less important than the duration of the procedure. Hassan et al [33] believed that an operation time of < 60 min had a better neurological outcome, and reported that the puncture to recanalization time was a significant predictor of unfavorable clinical outcomes. This was consistent with our analysis of a good outcome in patients using tirofiban (median 61 min versus mean 60 min). Vascular tortuosity, clot consistency, thrombectomy techniques, and other factors are known to affect operation time [34]. Several studies have shown that the non-modifiable patient factors of age and basic NIHSS score are important factors affecting prognosis [35–37], and age and NIHSS score were also important components of our nomogram.

In China, large vessel reocclusion, perforating branch occlusion, and microcirculation disorders may occur in people with ischemic stroke caused by all causes. In our non-tirofiban group of patients with large vessel occlusion acute ischemic stroke undergoing endovascular thrombectomy, we found that the stroke etiology was one of the important factors affecting functional outcomes (aOR 1.39; 95% CI 1.053–1.836; *p* = 0.02). However, in our intravenous tirofiban research, we did not find an effect of stroke type on functional outcomes, which may be due to the improvement of microcirculation disorders after intravenous tirofiban treatment. In Asia populations, ischemic stroke caused by intracranial atherosclerosis accounts for 30–50% [38]. And in the post hoc analysis of the RESCUE BT study found tirofiban with EVT improved 90-day outcome for patients with large vessel occlusion stroke due to intracranial atherosclerosis [39]. In the real world, tirofiban was a commonly used drug for rescue therapy and thrombosis during the perioperative period. Our research is of great clinical significance in exploring the potential characteristics of populations that can benefit from tirofiban treatment.

This study has several limitations that warrant discussion. First, although we performed a subgroup analysis based on a large-scale, placebo-controlled, double-blind database, we lacked data to validate our prediction model, which should be verified in future studies. Second, although we tried to include as many research parameters as possible, such as collateral circulation, infarction volumes, and the influence of postprocedural factors, they were not included in our analysis. Finally, we only included patients who received tirofiban with thrombectomy based on acute large-vessel occlusion, which may have biased the results.

## Conclusions

In this study, multivariate analysis revealed that younger age, lower serum glucose level, lower baseline NIHSS score, fewer total passes, shorter punctures to recanalization time, and mTICI scores of 2b to 3 were independent predictors of good outcomes after intravenous tirofiban with endovascular thrombectomy for large vessel occlusion stroke, all of which served as the basis for our nomogram, which showed an acceptable model fit and relatively good performance. However, further research is required to confirm the reliability of this model in other populations.

### Electronic supplementary material

Below is the link to the electronic supplementary material.

Supplementary Figure S1. The mediating role of sICH in the intravenous tirofiban with endovascular thrombectomy for large vessel occlusion stroke between baseline ASPECTS and good outcome. DE, direct effect; IE, indirect effect.

Supplementary Material 2

Supplementary Figure S2. The ROC curves for continuous variables in multivariate analysis of tirofiban alone combined with endovascular thrombectomy for large vessel occlusive stroke: A. ROC curve of Age, B. ROC curve of baseline NIHSS, C. ROC curve of serum glucose, D. ROC curve of total passes, E. ROC curve of puncture to recanalization time. AUC, the area under the curve; ROC, receiver operating characteristic.

Supplementary Material 4

Supplementary Figure S3. The nomogram for predicting a good outcome in patients with no-Tirofiban with endovascular thrombectomy for large vessel occlusion stroke. For each patient, we added the scores of the six influencing factors corresponding to the “Points”, and then the prediction results were obtained based on the predicted values corresponding to the “Total Points”. NIHSS, National Institutes of Health Stroke Scale; ASPECTS, Acute Stroke Prognosis Early Computed Tomography Score; mTICI, modified Thrombolysis in Cerebral Infarction score 2b (50%?99% reperfusion) to 3 (complete reperfusion).

## Data Availability

No datasets were generated or analysed during the current study.

## Declarations

### CONSORT guidelines

Our study adheres to CONSORT guidelines.
