# Supplementary figures and images for: Predictors of outcome in large vessel occlusion stroke patients with intravenous tirofiban treatment: a post hoc analysis of the RESCUE BT clinical trial
Source: BMC Neurol. 2024 Jul 1;24:227. doi: 10.1186/s12883-024-03733-w (PMC11218210; doi:10.1186/s12883-024-03733-w)

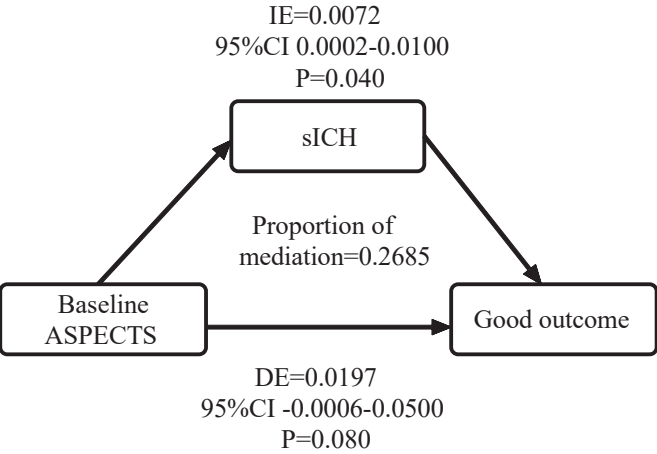

Supplement: Supplementary file 1 — Supplementary Figure S1. The mediating role of sICH in the intravenous tirofiban with endovascular thrombectomy for large vessel occlusion stroke between baseline ASPECTS and good outcome. DE, direct effect; IE, indirect effect. [file 12883_2024_3733_MOESM1_ESM.pdf]

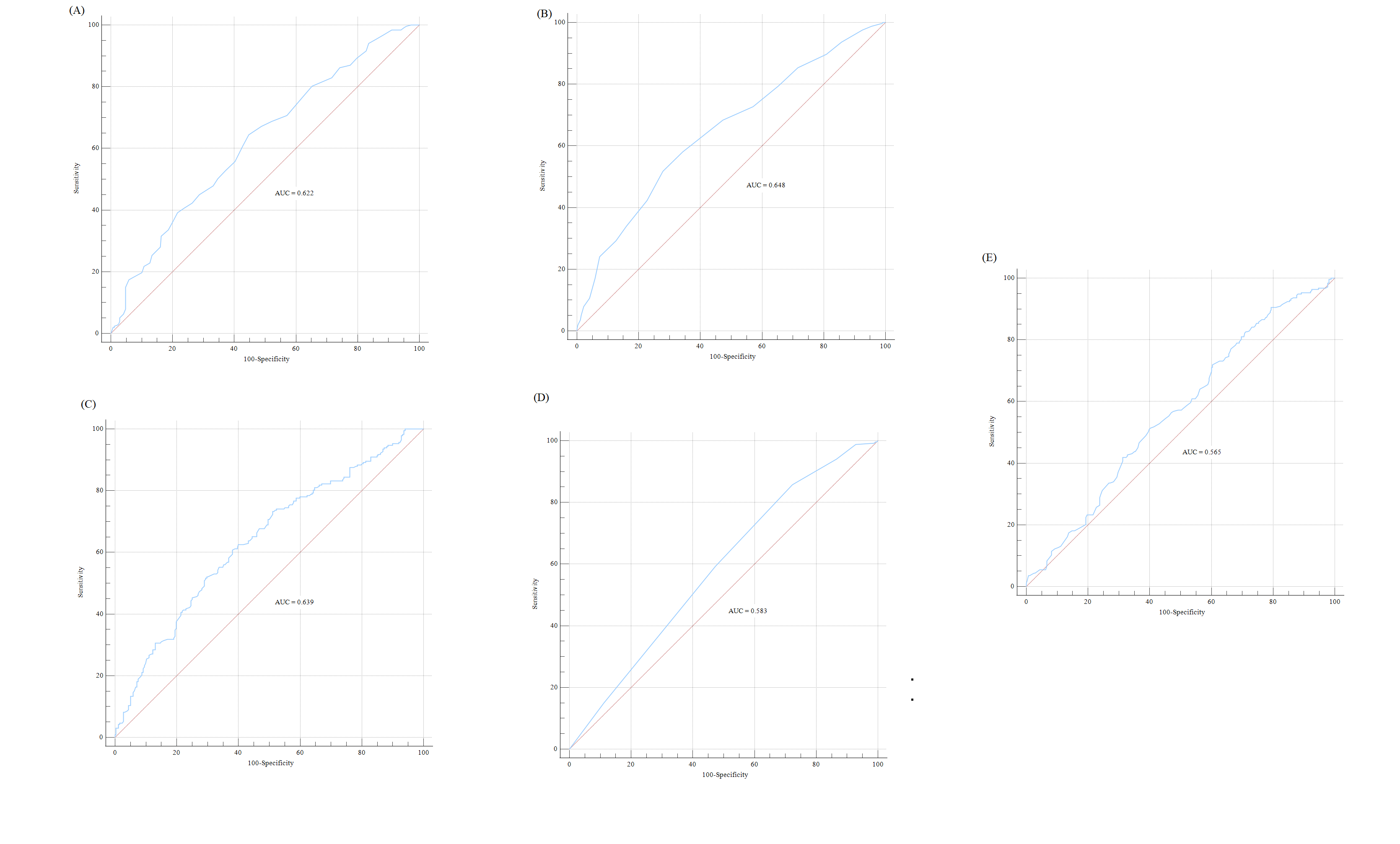

Supplement: Supplementary file 3 — Supplementary Figure S2. The ROC curves for continuous variables in multivariate analysis of tirofiban alone combined with endovascular thrombectomy for large vessel occlusive stroke: A. ROC curve of Age, B. ROC curve of baseline NIHSS, C. ROC curve of serum glucose, D. ROC curve of total passes, E. ROC curve of puncture to recanalization time. AUC, the area under the curve; ROC, receiver operating characteristic. [file 12883_2024_3733_MOESM3_ESM.png]

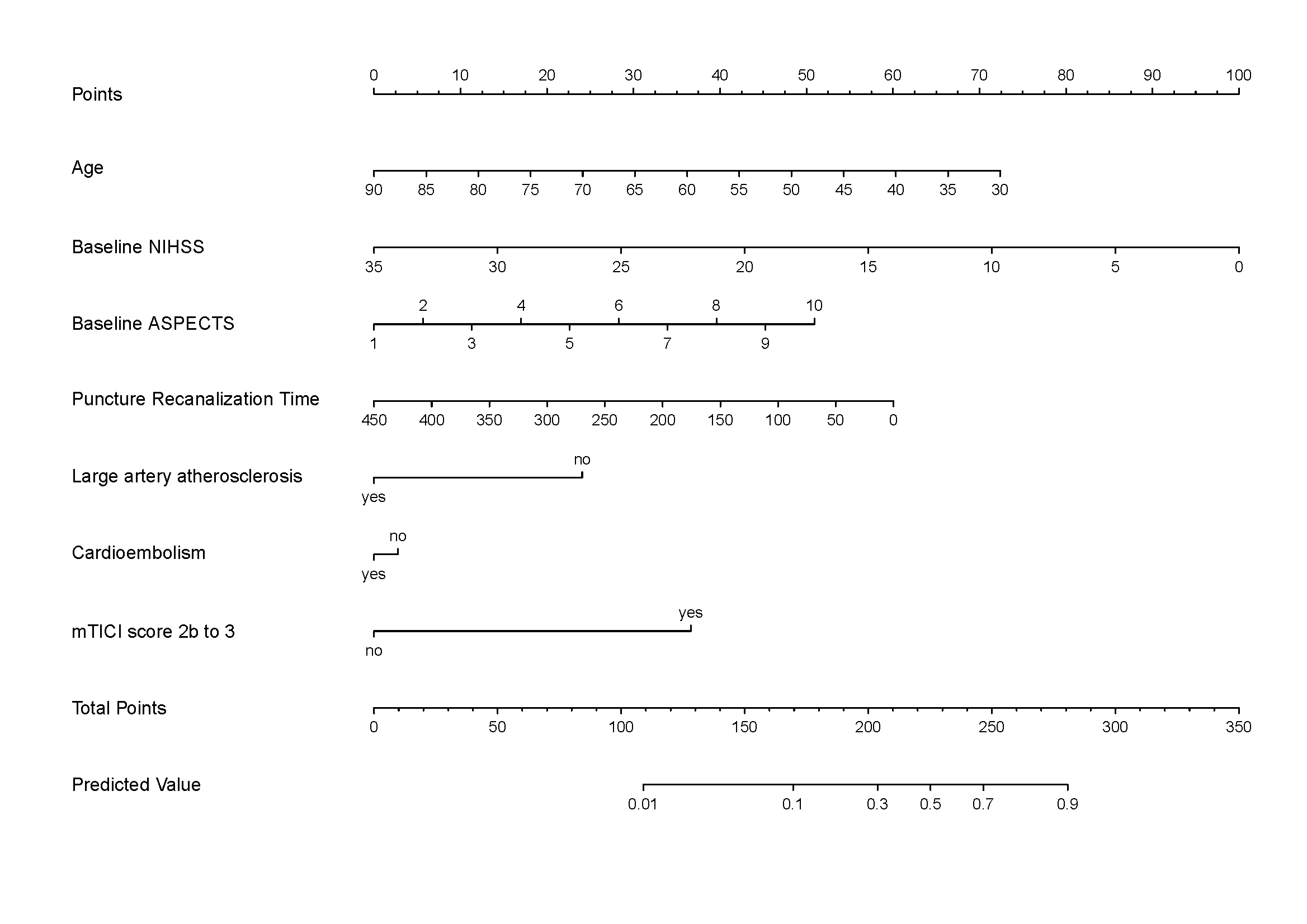

Supplement: Supplementary file 5 — Supplementary Figure S3. The nomogram for predicting a good outcome in patients with no-Tirofiban with endovascular thrombectomy for large vessel occlusion stroke. For each patient, we added the scores of the six influencing factors corresponding to the “Points”, and then the prediction results were obtained based on the predicted values corresponding to the “Total Points”. NIHSS, National Institutes of Health Stroke Scale; ASPECTS, Acute Stroke Prognosis Early Computed Tomography Score; mTICI, modified Thrombolysis in Cerebral Infarction score 2b (50%?99% reperfusion) to 3 (complete reperfusion). [file 12883_2024_3733_MOESM5_ESM.png]
