# Supplementary material for: Predictors of outcome in large vessel occlusion stroke patients with intravenous tirofiban treatment: a post hoc analysis of the RESCUE BT clinical trial
Source: BMC Neurol. 2024 Jul 1;24:227. doi: 10.1186/s12883-024-03733-w (PMC11218210; doi:10.1186/s12883-024-03733-w)
Supplement: Supplementary file 2 — Supplementary Material 2 [file 12883_2024_3733_MOESM2_ESM.doc]

| **Table S1. The VIF of baseline characteristics p<0.001** | | |
| --- | --- | --- |
| Characteristic | P value | VIF |
| Age | <0.001 | 1.236 |
| Sex | 0.014 | 1.104 |
| Baseline NIHSS | <0.001 | 1.101 |
| Baseline ASPECTS | 0.042 | 1.050 |
| SBP | 0.006 | 1.989 |
| DBP | 0.066 | 1.888 |
| Serum glucose | <0.001 | 1.504 |
| Vascular risk factor |  |  |
| Hypertension | 0.003 | 1.183 |
| Diabetes mellitus | <0.001 | 1.502 |
| Puncture to recanalization Time | 0.01 | 1.145 |
| Total passes | 0.001 | 1.103 |
| mTICI score 2b to 3 | <0.001 | 1.111 |

| **Table S2. Baseline characteristics of patients with and without sICH** | | | | |
| --- | --- | --- | --- | --- |
| Characteristic | With sICH | Without sICH | P value | |
| No. of patients | 49 | 471 |  | |
| Age, median (IQR), y | 71.0 [64.0, 74.0] | 67.0 [56.0, 74.0] | | 0.046 |
| Sex |  |  | | 0.987 |
| Female, no. (%) | 21 (42.9) | 196 (41.6) | |  |
| Male, no. (%) | 28 (57.1) | 275 (58.4) | |  |
| Baseline NIHSS, median (IQR) | 17.0 [12.0, 20.0] | 15.0 [11.5, 19.0] | | 0.147 |
| Baseline ASPECTS, median (IQR) | 7.0 [6.0, 8.0] | 8.0 [7.0, 9.0] | | 0.007 |
| SBP, mean (SD), mm Hg | 148.4 (27.3) | 146.1 (24.1) | | 0.528 |
| DBP, median (IQR), mm Hg | 84.0 [75.0, 92.0] | 83.0 [75.0, 94.0] | | 0.918 |
| Serum glucose, median (IQR), mmol/L | 7.4 [6.2, 10.6] | 6.9 [5.7, 8.5] | | 0.057 |
| Platelet count, median (IQR), 10^9/L | 162.0 [137.0, 206.0] | 201.0 [160.0, 240.2] | | 0.001 |
| Vascular risk factor |  |  | |  |
| Coronary heart disease, no. (%) | 6 (12.2) | 68 (14.4) | | 0.839 |
| Atrial fibrillation, no. (%) | 21 (42.9) | 149 (31.6) | | 0.152 |
| Hypertension, no. (%) | 25 (51.0) | 261 (55.4) | | 0.662 |
| Hyperlipidemia, no. (%) | 5 (10.2) | 78 (16.6) | | 0.341 |
| Diabetes mellitus, no. (%) | 13 (26.5) | 101 (21.4) | | 0.524 |
| Ischemic stroke, no. (%) | 6 (12.2) | 71 (15.1) | | 0.749 |
| Smoking, no. (%) | 8 (16.3) | 107 (22.7) | | 0.398 |
| Prestroke mRS score, no. (%) |  |  | | 0.984 |
| 0 | 46 (93.9) | 438 (93.0) | |  |
| 1 | 2 (4.1) | 20 (4.2) | |  |
| 2 | 1 (2.0) | 12 (2.5) | |  |
| 4 | 0 (0.0) | 1 (0.2) | |  |
| Stroke etiology, no. (%) |  |  | | 0.182 |
| LAA | 18 (36.7) | 232 (49.3) | |  |
| CE | 26 (53.1) | 187 (39.7) | |  |
| Other causes | 5 (10.2) | 52 (11.0) | |  |
| Occlusion sites, no. (%) |  |  | |  |
| Intracranial ICA | 19 (38.8) | 85 (18.0) | | 0.002 |
| M1 middle cerebral artery segment | 24 (49.0) | 327 (69.4) | |  |
| M2 middle cerebral artery segment | 6 (12.2) | 59 (12.5) | |  |
| Onset-Puncture Time, min, median (IQR) | 400.0 [267.0, 655.0] | 420.0 [280.0, 645.0] | | 0.877 |
| Puncture-recanalization Time, min, median (IQR) | 75.0 [45.0, 115.0] | 68.0 [41.0, 105.0] | | 0.506 |
| Total passes, median (IQR) | 2.0 [1.0, 3.0] | 1.0 [1.0, 2.0] | | 0.163 |
| mTICI score, no. (%) |  |  | |  |
| 0-2a | 6 (12.2) | 32 (6.8) | | 0.268 |
| 2b-3 | 43 (87.8) | 439 (93.2) | |  |
