# Supplementary material for: Predictors of outcome in large vessel occlusion stroke patients with intravenous tirofiban treatment: a post hoc analysis of the RESCUE BT clinical trial
Source: BMC Neurol. 2024 Jul 1;24:227. doi: 10.1186/s12883-024-03733-w (PMC11218210; doi:10.1186/s12883-024-03733-w)
Supplement: Supplementary file 4 — Supplementary Material 4 [file 12883_2024_3733_MOESM4_ESM.docx]

| **Table S3. Baseline characteristics of patients of no-Tirofiban Before Endovascular Thrombectomy** | | | |
| --- | --- | --- | --- |
|  | Good outcome | Poor outcome | P value |
| Characteristic | mRS 0-2 | mRS 3-6 |  |
| No. of patients | 194 | 233 |  |
| Age, median (IQR), y | 64.0 (55.0, 73.0) | 70.0 (63.0, 77.0) | <0.001 |
| Sex |  |  | 0.272 |
| Female, no. (%) | 73 (37.6) | 101 (43.3) |  |
| Male, no. (%) | 121 (62.4) | 132 (56.7) |  |
| Baseline NIHSS, median (IQR) | 14.0 (10.0, 18.0) | 17.0 (13.0, 20.0) | <0.001 |
| Baseline ASPECTS, median (IQR) | 8.0 (7.0, 9.0) | 7.0 (6.0, 9.0) | 0.001 |
| SBP, mean (SD), mm Hg | 142.6 (24.2) | 147.5 (23.1) | 0.034 |
| DBP, median (IQR), mm Hg | 83.5 (74.0, 95.0) | 84.0 (78.0, 95.0) | 0.322 |
| Serum glucose, median (IQR), mmol/L | 6.5 (5.6, 8.0) | 7.3 (6.0, 8.9) | 0.001 |
| Vascular risk factor |  |  |  |
| Coronary heart disease, no. (%) | 26(13.4) | 58 (24.9) | 0.004 |
| Atrial fibrillation, no. (%) | 61 (31.4) | 82 (35.2) | 0.475 |
| Hypertension, no. (%) | 92 (47.4) | 145 (62.2) | 0.003 |
| Hyperlipidemia, no. (%) | 24 (12.4) | 25 (10.7) | 0.706 |
| Diabetes mellitus, no. (%) | 32 (16.5) | 58 (24.9) | 0.046 |
| Ischemic stroke, no. (%) | 28 (14.4) | 56 (24.0) | 0.018 |
| Smoking, no. (%) | 54 (27.8) | 52 (22.3) | 0.23 |
| Prestroke mRS score, no. (%) |  |  | 0.013 |
| 0 | 183 (94.3) | 198 (85.0) |  |
| 1 | 8 (4.1) | 28 (12.0) |  |
| 2 | 3 (1.5) | 5 (2.1) |  |
| 3 | 0 (0.0) | 2 (0.9) |  |
| Stroke etiology, no. (%) |  |  | 0.01 |
| LAA | 69 (35.6) | 115 (49.4) |  |
| CE | 96 (49.5) | 97 (41.6) |  |
| Other causes | 29 (14.9) | 21 (9.0) |  |
| Occlusion sites, no. (%) |  |  | 0.155 |
| Intracranial ICA | 33 (17.0) | 57 (24.5) |  |
| M1 middle cerebral artery segment | 124 (63.9) | 139 (59.7) |  |
| M2 middle cerebral artery segment | 37 (19.1) | 37 (15.9) |  |
| Onset to puncture time, min, median (IQR) | 377.0 (240.0, 609.2) | 382.0 (245.0, 588.0) | 0.777 |
| Puncture to recanalization time, min, median (IQR) | 58.5 (37.0, 97.8) | 71.0 (47.0, 115.0) | 0.001 |
| Total passes^a^, median (IQR) | 2.0 (1.0, 2.0) | 2.0 (1.0, 3.0) | 0.405 |
| mTICI score 2b to 3, no. (%) | 187 (96.4) | 197 (84.5) | <0.001 |

Abbreviations: NIHSS, National Institutes of Health Stroke Scale; ASPECTS, Acute Stroke Prognosis Early Computed Tomography Score; SBP, systolic blood pressure; DBP, diastolic blood pressure; mRS, modified Rankin Scale; LAA, large artery atherosclerosis; CE, cardioembolism; mTICI, modified Thrombolysis in Cerebral Infarction score 2b (50%–99% reperfusion) to 3 (complete reperfusion).

^a^ The number of retriever total passes.

**Table S4. Multivariable analysis: predictors of a good outcome**

| Variable |  | aOR (95% CI) | p Value |
| --- | --- | --- | --- |
| Age |  | 0.964 (0.943-0.985) | 0.001 |
| Baseline NIHSS |  | 0.912 (0.869–0.957) | <0.001 |
| Baseline ASPECTS |  | 1.245 (1.064–1.457) | 0.006 |
| Stroke etiology |  | 1.390 (1.053–1.836) | 0.020 |
| Puncture to Recanalization Time |  | 0.994 (0.989–0.999) | 0.012 |
| mTICI score 2b to 3 |  | 5.034 (1.805–14.036) | 0.002 |
